# Domestic Correspondence

**Published:** 1890-08

**Authors:** 


### Domestic Correspondence.

---

To the Editor:

At the meeting of the Michigan Dental Association, Dr. Crouse presented the following views on the Dental Protective Association. Dr. Crouse's remarks were: First, that by combining in some such an organization as the Dental Protective Association we band the strength of ten thousand men into one,—all defence necessary can be made with but little more expense than would be required for an individual. The preparation of our case will answer for all,—all evidence can be collected better by an association than in any other way. Second, that it is very important that all get into the Association at this time, for the reason that an appealed suit, to the Supreme Court, before the formation of the Protective Association, will probably be decided in favor of the Crown Company, owing to deficiency of evidence and imperfect presentation. If the Crown Company win this suit, they will send notices all over the United States, thereby demoralizing the profession and causing many to pay them money who should be in the Protective Association, and thus avoid that calamity. The Protective Association will be ready with new evidence of a new record and can take care of its members against any claims of the Crown Company. If you are not in the Association, it will not take care of you when the time comes.

If each man in the profession will pay $10.00 and assume a responsibility of ten more, without any further assessments, we will have an organization that is sure to break up all this abuse, and save the dental profession an annual outlay, on an average, of $100.00 each, to unjust claimants.

Remember, it will cost more than $10.00 to join later, and will certainly cost more than that if you do not protect yourself.

*Resolved*, That the Michigan Dental Association heartily approves the aim and plan of the Dental Protective Association; and that it is further

*Resolved*, That it is the duty of every member of the dental profession, in this State, to join the Dental Protective Association. It is also

*Resolved*, That Dr. Crouse be requested to furnish the dental journals with an abstract of his remarks for publication.

W. H. Dorraner,
W. G. Saunders,
—— Douglas,
*Committee.*
Wm. Cleland,
*Secretary Michigan Dental Association.*

---

To the Editor:

The Virginia State Dental Association will hold its Twenty-first Annual Session in the high-school building, at Roanoke, Virginia, Tuesday, August 26, 1890, beginning at nine o'clock A.M.

We confidently expect this to be the largest and best meeting the Association has ever held.

All members of the profession are invited to attend, and will receive a cordial welcome.

J. Hall Moore,
*Corresponding Secretary.*

Richmond, Va., July 11, 1890.

---

The State Board of Dental Examiners will meet at the same time and place for the examination of candidates to practice dentistry in the State. All applicants must be graduates of some reputable dental college.

W. E. Norris,
*Secretary.*

